# Development of associations between elementary school students’ mindsets and attentional neural processing of feedback in an arithmetic task

**DOI:** 10.3389/fpsyg.2023.1155264

**Published:** 2023-03-14

**Authors:** Ita Puusepp, Tanja Linnavalli, Tuisku Tammi, Minna Huotilainen, Teija Kujala, Sonja Laine, Elina Kuusisto, Kirsi Tirri

**Affiliations:** ^1^Faculty of Educational Sciences, University of Helsinki, Helsinki, Finland; ^2^Cognitive Brain Research Unit, Faculty of Medicine, University of Helsinki, Helsinki, Finland; ^3^Centre of Excellence in Music, Mind, Body and Brain, University of Helsinki, Helsinki, Finland; ^4^Cognitive Science, Department of Digital Humanities, University of Helsinki, Helsinki, Finland; ^5^Viikki Normal School, University of Helsinki, Helsinki, Finland; ^6^Faculty of Education and Culture, Tampere University, Tampere, Finland

**Keywords:** mindset, P300 - event related potential, math, feedback, implicit beliefs

## Abstract

The aim of this study was to examine the development of the associations between elementary school students’ mindsets and the attentional neural processing of positive and negative feedback in math. For this, we analyzed data collected twice from 100 Finnish elementary school students. During the autumn semesters of their 3rd and 4th grade, the participants’ general intelligence mindset and math ability mindset were measured with a questionnaire, and their brain responses elicited by performance-relevant feedback were recorded during an arithmetic task. We found that students’ fixed mindsets about general intelligence and math ability were associated with greater attention allocated to positive feedback as indicated by a larger P300. These associations were driven by the effects of mindsets on attention allocation to positive feedback in grade 4. Additionally, 4th graders’ more fixed general intelligence mindset was marginally associated with greater attention allocated to negative feedback. In addition, the effects of both mindsets on attention allocation to feedback were marginally stronger when the children were older. The present results, although marginal in the case of negative feedback and mainly driven by effects in grade 4, are possibly a reflection of the greater self-relevance of feedback stimuli for students with a more fixed mindset. It is also possible that these findings reflect the fact that, in evaluative situations, mindset could influence stimulus processing in general. The marginal increase in the effects of mindsets as children mature may reflect the development of coherent mindset meaning systems during elementary school years.

## 1. Introduction

Encountering feedback—information about one’s performance or understanding—is part of students’ daily experience in school. The effect of feedback on student outcomes, though, is far from homogeneous (Wisniewski et al., 2020) and seems, at least partly, to depend on individuals’ beliefs about the malleability of their abilities (Hu et al., 2017). Mindsets—core beliefs about the malleability of human abilities—can be understood as meaning systems that perform an organizing function regarding the interpretation of one’s experiences (e.g., receiving negative feedback about one’s performance; Hong et al., 1999; Molden and Dweck, 2006). In line with this, mindsets have been associated with perceptions of negative feedback (Zingoni and Byron, 2017). The associations between students’ mindsets and their perceptions of and reactions to feedback, errors, and failure experiences have been investigated using questionnaires (e.g., Zingoni and Byron, 2017), behavioral measures (e.g., Janssen et al., 2022), and also qualitative data from interviews (e.g., Limeri et al., 2020). Neuroscientific research provides the opportunity to gain a better understanding of mindset meaning systems by enabling the inspection of the neural processes associated with the perception and cognition of feedback, which is unattainable using behavioral measures or surveys. Nevertheless, such neuroscientific studies on mindsets are scarce. To our knowledge, there are two studies on the associations between mindset and automatic attention allocation to feedback (Mangels et al., 2006; Puusepp et al., 2021), both of them cross-sectional research. The tentative findings of these studies require replication. Furthermore, studies suggest that elementary school years are a period during which students’ mindsets as meaning systems develop (Kinlaw and Kurtz-Costes, 2007; Gunderson et al., 2018), which highlights the importance of longitudinal studies among this age group. Therefore, the aim of the present study was to extend the previous research by investigating the development of the associations between mindsets and the attentional neural processing of feedback among elementary school students. More specifically, we focused on students’ general intelligence and math ability mindsets and their attentional neural processing of positive and negative performance-relevant feedback in math in two consecutive school years.

Mindsets refer to core beliefs that people hold about the nature and malleability of certain human abilities and attributes, such as intelligence or personality (Dweck, 2000). These beliefs exist on a spectrum from a fixed mindset, believing that human abilities are unchangeable and fixed (entity theory), to a growth mindset, believing that these qualities and attributes are malleable and can be improved with effort and adaptive strategies (incremental theory; Dweck, 2000). Mindsets, while being mostly implicit, form an intricate meaning system that performs an organizing function regarding the interpretation of one’s experiences and the planning of future behavior (Hong et al., 1999). The relevance of mindsets in educational contexts has been demonstrated among learners at various educational levels (Tempelaar et al., 2015; Gunderson et al., 2018; Bostwick et al., 2020). More specifically, a growth mindset has been shown to be associated with learning goals and mastery-oriented strategies (Burnette et al., 2013) as well as better academic outcomes (Claro and Loeb, 2019) and well-being (Alvarado et al., 2019). Mindsets have proven to be especially relevant in situations involving challenges and setbacks (Burnette et al., 2013). Namely, people with different beliefs about the malleability of abilities attribute difficulty or failure to different potential causes. While a fixed mindset leads one largely to attribute one’s mistakes and setbacks to a lack of fixed ability, a growth mindset leads one mainly to interpret one’s mistakes as an indicator of a lack of sufficient effort or the use of an ineffective strategy (Yeager and Dweck, 2012). Individuals can possess different mindsets for different domains, but research has nonetheless demonstrated a certain intraindividual generality for these beliefs across domains (Lewis et al., 2021).

It has been suggested that, during elementary school years, students’ mindset meaning systems develop towards greater coherence, as indicated by an increase in associations between mindsets and theoretically relevant constructs, such as goal-orientation (Kinlaw and Kurtz-Costes, 2007). Moreover, research suggests that even though students hold different mindsets about different domains during the first years of elementary school, these mindsets become self-relevant only once the students are older (Gunderson et al., 2017). More specifically, Gunderson et al. (2017) demonstrated that while students generally believed success in math required more fixed ability than did success in reading and writing, younger students believed that this only concerned the success of adults rather than that of their peers. In addition, other studies indicate that people tend to hold a stronger fixed mindset about math than about some other subject domains (e.g., Leslie et al., 2015; Heyder et al., 2020). Additionally, math is one of the subjects that many students consider the most important in school (Dundar and Rapoport, 2014; McGeown and Warhurst, 2020). Math programs become highly cognitively demanding for students after the first years of elementary school (Tsang et al., 2015), and consequently mindsets are especially important during this period. Furthermore, in Finland, where the present study was conducted, notable increases have been shown in students’ negative attitudes towards math (disliking math and low math self-efficacy) during elementary and middle school years (Tuohilampi and Hannula, 2013), and concerns about declining math achievement have also been raised (Metsämuuronen and Nousiainen, 2021). Therefore, in the present study, we used an arithmetic task to focus on students’ reactions to feedback in the domain of math. As to mindsets, we assessed both general intelligence mindsets and math ability mindsets to examine their subject-domain specificity regarding associations with the attentional neural processing of feedback.

In line with the theory of mindsets as meaning systems that perform an organizing function regarding the interpretation of experiences (Hong et al., 1999), research has demonstrated that mindsets are associated with error-monitoring (Moser et al., 2011; Schroder et al., 2014, 2017) and the attentional neural processing of feedback (Mangels et al., 2006; Puusepp et al., 2021). While automatic allocation of attentional resources to the stimulus cannot be assessed through behavioral or survey measures, event-related potentials (ERPs) provide a covert measure of these processes. ERPs are fluctuations of voltage recorded using an electroencephalogram (EEG) that are time-locked to a certain event (e.g., presentation of a stimulus) or response execution (e.g., the pressing of a button; Woodman, 2010; Kappenman and Luck, 2011).

One of the most frequently examined ERP components elicited by feedback is P300. P300 is a positive-going waveform reflecting the processing of attention-demanding stimulus in general; thus, it is not limited to feedback-stimulus processing (for a review, see Polich, 2007). This waveform peaks at approximately 300–600 ms after the appearance of the eliciting stimulus, and it is assumed to reflect attentional processes and to signal unexpected changes that are relevant for behavioral adjustment. A larger P300 amplitude is associated with greater availability or allocation of attentional resources for processing the stimulus, while a smaller amplitude indicates the reverse (Polich, 2007). As an increase in P300 amplitude reflects greater attention allocation, it has also been associated with the heightened psychological significance of certain stimuli when compared to more neutral stimuli (Gray et al., 2004). In the context of reward and feedback processing, unexpected stimuli or outcomes have been observed to elicit a larger P300 than that produced by anticipated stimuli or outcomes (Hajcak et al., 2005; Wu and Zhou, 2009; for a review, see San Martín, 2012). Furthermore, the size of the outcome (either loss or reward) has been associated with the feedback-related P300 amplitude, with outcomes of a greater magnitude relating to a larger P300 than that elicited by smaller outcomes (San Martín, 2012). Nonetheless, the findings on the effect of feedback or outcome valence on P300 have been somewhat inconsistent, with some studies reporting feedback-related P300 to be greater in the case of positive feedback (adults: Wu and Zhou, 2009; Ernst and Steinhauser, 2012), others finding a larger P300 for negative feedback (children: Arbel, 2020; adults: Butterfield and Mangels, 2003), and some showing it to be insensitive to the valence of the feedback stimulus (children: Ferdinand et al., 2016; Du et al., 2018; adults: Hajcak et al., 2005; Yeung et al., 2005; for a review, see San Martín, 2012). Furthermore, the findings of Buritica et al. (2018) indicate that, in children, differences between the positive- and negative-feedback P300 depend on the task design. Attentional neural processing of feedback has also been shown to associate with learning from corrective feedback, with a greater feedback-related P300 elicited when tasks initially answered incorrectly were later answered correctly than when such tasks were unsuccessfully answered in a retest (Ernst and Steinhauser, 2012).

Another ERP associated with mindset is error-related positivity (Pe). Pe is a positive-going ERP that is elicited on error commission in reaction time tasks peaking, in general, between 200 and 400 ms after an erroneous button press. Pe indicates awareness of committing an error and is assumed to reflect the motivational significance of that error (Overbeek et al., 2005). Pe and P300 are assumed to reflect similar processes—conscious processing of motivationally significant events (Ridderinkhof et al., 2009). Thus, results demonstrating that a greater difference in Pe between erroneous and correct trials is associated with a growth mindset (Moser et al., 2011; Schroder et al., 2017) are in line with the theory of mindsets as meaning systems: for growth-minded individuals, mistakes are motivationally significant for enhancing their ability, as such errors are an indicator of the need for more effort or different strategies (Molden and Dweck, 2006). To our knowledge, there are four studies that have explored the association between mindsets and automatic attention allocation to mistakes. Two of these studies have found a growth mindset to be associated with greater attention allocation to committed errors (compared to correct responses), as indicated by a larger difference between Pe amplitudes for erroneous and correct trials (adults: Moser et al., 2011; school-aged children: Schroder et al., 2017). In their study with experimentally induced growth and fixed mindsets, Schroder et al. (2014) nonetheless produced somewhat different results: a smaller Pe in the growth mindset group compared to the fixed mindset group. Moreover, they found that participants with an induced growth mindset exhibited smaller Pe amplitudes elicited by not only erroneous, but also correct responses. Recently Janssen et al. (2021) suggested that the associations between mindset and Pe found in previous studies might have been confounded with the P300 response elicited by the stop-stimulus in the experimental tasks used. Namely, they found a more fixed mindset to be marginally associated with a larger P300 elicited by the stop-stimulus in a go/no-go task. These results suggest that mindsets might be associated with stimulus processing more generally and not only with error monitoring.

In addition to the aforementioned studies on mindsets and error monitoring, to our knowledge there are two studies that have explored associations between mindsets and the attentional neural processing of feedback (Mangels et al., 2006; Puusepp et al., 2021). Error monitoring and feedback processing could be considered similar processes, with error monitoring requiring internal feedback (it is the person themselves who realizes they have made a mistake, without any external feedback), and feedback processing requiring external feedback. Based on the study by Mangels et al. (2006) on adults, a fixed mindset about intelligence, when compared to a growth mindset, was associated with a larger anterior frontal P300 elicited by negative performance-relevant feedback in a general knowledge test. As this larger P300 was also associated with the endorsement of performance goals, it was assumed to reflect heightened attention to evaluative performance-relevant feedback among fixed-minded participants. In turn, Puusepp et al. (2021) explored the associations between domain-general and domain-specific mindsets and feedback processing among elementary school students. They found a marginally significant unique association between math ability mindsets and the difference between the P300 elicited by negative and positive feedback in an arithmetic task, with general intelligence mindsets demonstrating no such association. Nevertheless, in that study, only the difference between the ERPs elicited by positive and negative feedback was explored, with no inspection of the associations between mindsets and the processing of positive and negative feedback separately.

The current study is part of the project Copernicus—Changing Mindsets about Learning: Connecting Psychological, Educational and Neuroscientific Evidence. This project uses a multidisciplinary approach to investigate the mindsets of elementary school students (Laine et al., 2022) and their parents (Levinthal et al., 2021) as well as the beliefs and pedagogical practices of teachers (Rissanen et al., 2019). Additionally, the project aims to develop an intervention program to be used by teachers in Finland to support the development of their elementary school students’ growth mindsets (Rissanen et al., 2021). The main aim of the present study was to explore the development of the associations between elementary school students’ mindsets and the attentional neural processing of feedback. More specifically, we aimed to extend the earlier findings of Mangels et al. (2006) and Puusepp et al. (2021) by (a) inspecting the associations between mindset and attention allocation to performance-relevant feedback among children, (b) taking into account the subject-domain-specificity of mindsets regarding these associations, and (c) longitudinally exploring the development of these associations. Therefore, the current study explored the associations between elementary school students’ general intelligence mindset and math ability mindset and their P300 responses elicited by performance-relevant positive and negative feedback in an arithmetic task in two consecutive school years. Our main research questions and hypotheses were as follows:

RQ1. How are children’s mindsets (general intelligence mindset and math ability mindset) associated with their attention allocation to performance-relevant positive and negative feedback in an arithmetic task? We hypothesized that:

(H1a) mindsets are associated with the attentional neural processing of negative, but not positive, performance-relevant feedback (Mangels et al., 2006; Puusepp et al., 2021),

(H1b) the associations between math ability mindset and feedback-related P300 during an arithmetic task are stronger than the associations between general intelligence mindset and feedback-related P300 during an arithmetic task (Puusepp et al., 2021).

RQ2. How do associations between children’s mindsets (general intelligence mindset and math ability mindset) and attention allocation to performance-relevant feedback develop? We hypothesized that:

(H2) the association between mindset and feedback-related P300 strengthens as children age (Kinlaw and Kurtz-Costes, 2007).

## 2. Materials and methods

### 2.1. Participants

A total of 104 participants from two Finnish public elementary schools in the Helsinki metropolitan area were initially recruited for this study. One of the schools was situated in a low socioeconomic status (SES) area and the other in a medium SES area (Vilkama et al., 2014). Two outlier participants with P300 amplitudes (measured in grade 3 or 4) exceeding 3 standard deviations from the mean were excluded from the final sample. Participants with complete data from either both years or one year were included in the analyses, resulting in a final sample of 100 participants (50 girls, 45 boys, and 5 who responded “Other”; M_*age*_ at the beginning of the study = 8.94 years, SD_*age*_ = 0.42 years, Min_*age*_ = 8 years, Max_*age*_ = 11 years). Reasons for missing data included withdrawing from the study, absence from school on the data collection days, or a technical issue during the EEG-recording.

### 2.2. Measures

#### 2.2.1. Mindsets

For assessing participants’ *General Intelligence Mindset (GIM)*, four Entity Theory statements from the Implicit Theories of Intelligence Scale (Dweck, 2000) were used (e.g., “You have a certain amount of intelligence, and you cannot really do much to change it”). In order to assess participants’ *Math Ability Mindset (MAM)*, the same four Entity Theory statements from the Implicit Theories of Intelligence Scale used for assessing GIM were adapted to be math-specific. Participants indicated the degree to which they agreed with each of the statements by marking one of six circles, which varied in size (ranging from small to big) and were mapped to a 6-point scale (1—do not agree at all … 6—completely agree). Four average scores were used; GIM in grade 3 and 4 and MAM in grade 3 and 4. Higher scores indicate a greater endorsement of a growth mindset. The internal reliabilities of the mindset instruments from both years were good (Cronbach’s α ranged from 0.76 for GIM in grade 3 to 0.86 for MAM in grade 4).^1^

#### 2.2.2. Arithmetic task

**FIGURE 1:**
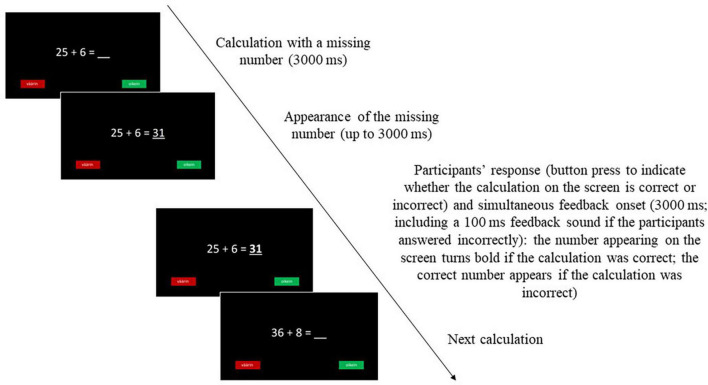
Sequence of events in a trial of the two-alternative choice arithmetic task.

The participants’ *P300 responses elicited by feedback* were recorded during the completion of an age-appropriate two-alternative choice arithmetic task (Figure 1). Each trial in the task consisted of an arithmetic calculation with one number missing; this calculation was presented at a central location on the computer screen for 3,000 ms. Subsequently, either a correct or incorrect answer was presented in the place of the missing number for a maximum of 3,000 ms. During this 3,000 ms response window, the participants were instructed to use their dominant hand to press one of the two buttons on the response box to indicate whether they thought the number appearing in the calculation was the correct or incorrect answer. If the number on the screen was correct, the participant’s response was followed by the correct answer, shown in bold on the monitor for 3,000 ms. If the number presented on the screen was incorrect, this incorrect answer changed into the correct answer, which was also displayed on the screen for 3,000 ms. If the participant responded incorrectly, their response was immediately followed by a feedback tone lasting 100 ms to ensure that the participant was aware of their mistake. If the participant failed to produce a response during the 3,000 ms response window, a time-out message appeared at the center of the screen for 3,000 ms before the calculation of the next trial appeared.

Before the arithmetic task, the participants completed a practice block consisting of five correct trials and five incorrect trials to ensure that they had understood the task. Based on their performance during the practice block, the participants were subsequently administered either an easier (0–5 trials answered correctly) or a more difficult version (6–10 trials answered correctly) of the actual task in order to ensure that the calculations in the task were sufficiently, but not overly, challenging for the participants.

The actual task consisted of two blocks (47 trials in the first block and 46 trials in the second block) with a total of 93 trials. The 93 trials (48 correct calculations and 45 incorrect calculations) were presented in random order to each participant. Between the blocks, the children were permitted a 5- to 10-min refreshment pause. In order to avoid possible motor response confounds in the aggregated data, the positions of the two buttons on the response box were alternated every second experimental day (Grootswagers et al., 2017). The difficulty of the calculations in the arithmetic tasks was adjusted according to the grade level, with the 3rd grade task including only addition and subtraction and 4th grade task also including multiplication and division.

### 2.3. Procedure

The children’s participation in the study was voluntary. Written parental consent for the study was obtained. The children and their parents were informed about the research procedures and their right to withdraw their participation at any moment of the study. The University of Helsinki Ethical Review Board reviewed and approved the research project for the study.

The students completed a questionnaire including the mindset scales as part of a longer questionnaire during their regular school activities in the autumn semester of their 3rd and 4th grade. The questionnaires were administered by a researcher who explained the questionnaire procedure with examples of statements with response options. For data collection in grade 3, the researcher read each question and response option aloud as the participants completed the electronic questionnaire on laptops or tablets provided by the school. In grade 4, the participants completed the electronic questionnaire individually during a school lesson. The procedure lasted approximately 40 min.

The psychophysiological recordings were performed by the experimenter(s) in a separate room at the school premises during regular school hours. Before the recordings, the children were briefed about how the experiment would proceed and reminded of their right to withdraw their participation at any moment. After the task and the recording, the children were compensated for their participation with sweets and stickers. The entire procedure lasted for 60-75 min per participant, with the duration of the EEG-recording being approximately 20 min per participant.

### 2.4. Data recording and processing

Continuous electroencephalographic activity was recorded with portable equipment (BrainVision QuickAmp amplifier) using 32 Ag-AgCl active electrodes (ActiCap, Brain Products, Germany). Electrolyte gel (Signa Gel, Bio-Medical Instruments, Inc., Warren, MI, USA) was used at each electrode. The data were recorded with a BrainVision Recorder at a 250 Hz sampling rate. The Recording reference was Fpz or FCz, depending on the size of the cap used. Afterwards, the EEG data were processed with MATLAB R2019a software (Mathworks, Natick, MA, USA) with the EEGLAB 19.0 toolbox. The signal was band-pass filtered with cutoffs of 0.1 and 30 Hz and segmented into epochs beginning 200 ms before each button press and continuing for 750 ms following each button press. In addition to visual inspection, artifactual epochs were rejected by detecting abnormal trends and abnormal spectra, and eye movement artefacts were removed using independent component analysis (Delorme and Makeig, 2004). Subsequently, the data were re-referenced to the mean of the mastoid electrodes.

**FIGURE 2:**
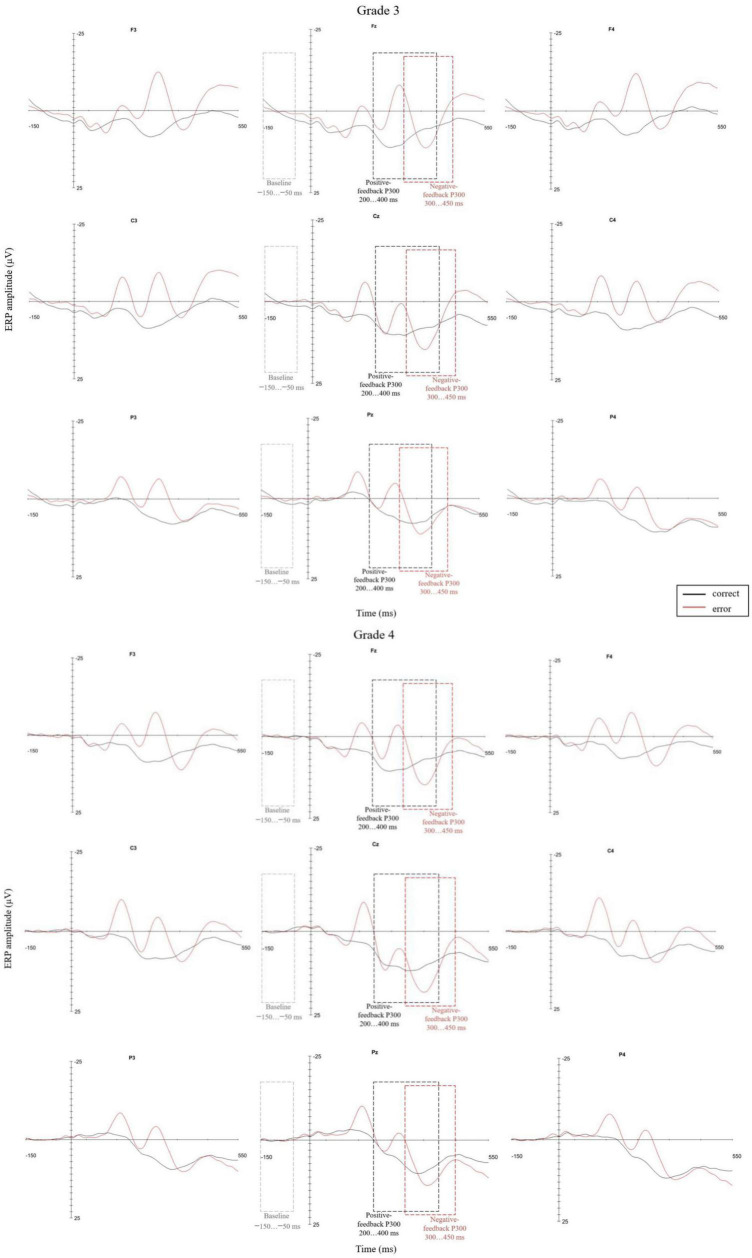
Feedback-locked waveforms for positive and negative feedback trials at both grade levels with indicated baseline and P300 time windows at frontal, central, and parietal electrodes. The 0 point on the time scale represents the feedback stimulus onset. The P300 amplitudes were collected based on individual peak latencies: positive-feedback P300 within the 200–400 ms and negative-feedback P300 within the 300–450 ms time window after feedback stimulus onset.

Feedback-locked P300 amplitudes were calculated relative to a -150 to -50 ms baseline window, which was also approximately 150–50 ms prior the response (button press), as the time difference between the button press and the feedback stimulus onset was only a few milliseconds. In order to obtain feedback-related ERPs regarding participants’ authentic decisions about the accuracy of the math calculations and in order to exclude trials involving accidental button presses, all trials where the reaction time was less than 300 ms after the appearance of the pre-response-stimulus (the answer appearing in place of the missing number of the equation on the screen) were omitted from the analyses (Thomas et al., 1981). In addition, time-out trials were excluded from further analyses. Furthermore, to ensure reliable averages for the ERPs, a minimum of six trials was considered necessary for each participant for both erroneous and correct trials (Pontifex et al., 2010). The average number of correct trials included in the further analyses was 42 (min 20, max 73) per participant in grade 3 and 41 (min 24, max 62) in grade 4. The average number of erroneous trials in grade 3 was 27 (min 6, max 53) per participant, while in grade 4 it was 24 (min 8, max 40) per participant. Subsequently, the averaged ERPs for correct and erroneous trials were visually inspected in order to determine the electrode sites with maximal amplitudes and to calculate the most relevant time windows for the correct- and erroneous trial P300s (Figure 2). Accordingly, feedback-locked grand average P300 amplitudes were calculated for three electrode sites along the scalp midline (Fz, Cz, and Pz). The positive-feedback P300 was calculated as the mean amplitude over a 50 ms time window around each participant’s average positive peak between latencies 200 and 400 ms after the onset of the positive feedback stimulus. The negative-feedback P300 was calculated as the mean amplitude over a 50 ms time window around each participant’s average positive peak between latencies 300 and 450 ms after negative feedback stimulus onset.

### 2.5. Overview of data analysis

The descriptive statistics of mindsets, behavioral variables, and P300 were calculated and the distribution of data inspected visually. None of the variables severely violated the normality assumption. Therefore, Pearson correlation was used to inspect the relationships between the study variables. The research questions were answered by using linear mixed modelling (LMM). R package *lme4* was used, and *p*-values were computed with the *lmerTest* package (Kuznetsova et al., 2017), using Satterthwaite’s method to estimate degrees of freedom. When statistically significant effects of fixed factors emerged in LMM, the *emmeans* package was used to compute the estimated marginal means, and Bonferroni-corrected post hoc comparisons were performed. When significant effects of continuous variables emerged in LMM, simple slope comparisons were used. To calculate effect sizes (partial epsilon squares), the *effectsize* package was used. The continuous variables were standardized for modeling, and thus standardized coefficients are reported.

Before conducting the main analysis, a model without any fixed predictors was first specified to estimate how much variance in P300 amplitude could be attributed to individual differences between participants within classrooms and between different classes (i.e., random effects of participant and class). As this model demonstrated a low variance of P300 to be explained by the nestedness of participants in classrooms, subsequent analyses were conducted without random effects of class. Second, the effects of background variables (grade level, feedback type, electrode site) and the behavioral measure of task accuracy on P300 were inspected using LMM (for the results of this LMM, see Supplementary Table 3). Based on these results, the main models were constructed, with grade level (3rd or 4th grade), feedback condition (positive or negative feedback) and electrode site (Fz, Cz, or Pz) used as within-subject fixed factors. Overall accuracy on the task and GIM or MAM scores were included as between-subject fixed continuous predictors. As to random effects in the main models on longitudinal data, (1) a random intercept by the participant and (2) feedback type, grade level and GIM or MAM as random slopes by the participant were included. In this way, random effects resulting from repeated measures regarding the same participants were accounted for in the models, allowing for between-subject variability. These models were inspected for potentially influential extreme values, normality of residuals, homoscedasticity and multicollinearity, with no violations of the assumptions being found.

## 3. Results

### 3.1. Mindsets

A range of mindset endorsements was observed, with participants’ GIM and MAM scores falling between fixed and growth extremes (Table 1). Positive correlations between GIM and MAM were observed both in grade 3 (*r* = 0.43, *p* < 0.001) and grade 4 (*r* = 0.67, *p* < 0.001; Table 2). Both GIM and MAM showed stability over the two years, as indicated by the within-construct correlations over the two measurement points (*r* = 0.22, *p* = 0.035; *r* = 0.35, *p* < 0.001, respectively).

**TABLE 1:** Descriptive statistics for study variables from both years.

Variable	*n*	*M (SD)*	*Min*	*Max*
1. Positive-fb P300 at Fz (μV) in grade 3	100	13.04 (12.66)	-19.58	51.96
2. Positive-fb P300 at Cz (μV) in grade 3	100	13.33 (13.04)	-15.62	48.15
3. Positive-fb P300 at Pz (μV) in grade 3	100	10.19 (13.38)	-16.30	40.64
4. Negative-fb P300 at Fz (μV) in grade 3	100	11.22 (14.61)	-37.83	56.89
5. Negative-fb P300 at Cz (μV) in grade 3	100	14.86 (14.29)	-23.85	49.83
6. Negative-fb P300 at Pz (μV) in grade 3	100	11.42 (12.13)	-24.85	47.96
7. Task accuracy (%) in grade 3	100	60.5 (10.5)	38.1	88.8
8. General intelligence mindset in grade 3	99	3.65 (1.20)	1.00	6.00
9. Math ability mindset in grade 3	99	4.15 (1.20)	1.25	6.00
10. Positive-fb P300 at Fz (μV) in grade 4	96	12.95 (8.36)	-6.85	34.44
11. Positive-fb P300 at Cz (μV) in grade 4	96	14.35 (10.26)	-10.32	38.32
12. Positive-fb P300 at Pz (μV) in grade 4	96	10.82 (10.19)	-9.45	35.22
13. Negative-fb P300 at Fz (μV) in grade 4	96	15.79 (11.39)	-10.25	57.85
14. Negative-fb P300 at Cz (μV) in grade 4	96	19.30 (13.22)	-6.26	55.78
15. Negative-fb P300 at Pz (μV) in grade 4	96	14.52 (10.91)	-11.16	43.54
16. Task accuracy (%) in grade 4	97	64.8 (9.5)	46.7	92.3
17. General intelligence mindset in grade 4	97	3.91 (1.26)	1.25	6.00
18. Math ability mindset in grade 4	97	4.36 (1.20)	1.00	6.00

fb, feedback.

**TABLE 2:** Correlations between study variables from grades 3 and 4.

Variable	1	2	3	4	5	6	7	8	9
1. Positive-fb P300 at Fz (μV)	**0**.25*	0.80***	0.62***	0.43***	0.45***	0.44***	0.00	-0.29**	-0.17
2. Positive-fb P300 at Cz (μV)	0.89***	**0**.38***	0.85***	0.32**	0.48***	0.48***	-0.14	-0.31**	-0.25*
3. Positive-fb P300 at Pz (μV)	0.72***	0.86***	**0**.46***	0.12	0.30**	0.47**	-0.28**	-0.27**	-0.27*
4. Negative-fb P300 at Fz (μV)	0.61***	0.48***	0.30**	**0**.32**	0.78***	0.61***	0.34***	-0.10	0.13
5. Negative-fb P300 at Cz (μV)	0.58***	0.57***	0.40***	0.81***	**0**.38***	0.83***	0.31**	-0.09	0.01
6. Negative-fb P300 at Pz (μV)	0.52***	0.57***	0.61***	0.63***	0.80***	**0**.20*	0.22*	-0.10	-0.05
7. Task accuracy (%)	-0.07	-0.17^†^	-0.24*	0.03	0.05	-0.08	**0**.58***	0.11	0.25*
8. General intelligence mindset	-0.05	-0.10	-0.17^†^	0.04	-0.02	-0.03	-0.02	**0**.22*	0.67***
9. Math ability mindset	0.05	-0.09	-0.22*	0.16	0.03	-0.09	0.10	0.43***	**0**.35***

fb, feedback. Correlations between variables in grade 3 are presented under the diagonal, while correlations between variables in grade 4 are shown above the diagonal. Within-construct correlations are presented in bold in the diagonal. ^†^*p* < 0.10; *p < 0.05; **p < 0.01; ***p < 0.001.

### 3.2. Behavioral data

In the 3rd grade, the participants were correct, on average, in 60.5% (SD = 10.5%) of the trials (excluding timed-out trials), with the average accuracy in the group of students completing the easier version of the task (*n* = 38) being 57.4% (SD = 8.2%) and the accuracy for the more difficult version (*n* = 62) being 62.4% (SD = 11.4%). In the 4th grade, the average task accuracy was 64.8% (SD = 9.5%), with the average accuracy in the group of students completing the easier version of the task (*n* = 34) being 64.0% (SD = 8.4%) and the accuracy for the more difficult version (*n* = 63) being 65.3% (SD = 10.0%). Regarding associations with mindset, a higher MAM was associated with greater task accuracy in grade 4 (*r* = 0.25, *p* = 0.015), while this association was not significant in grade 3 (Table 2).

### 3.3. Feedback-related P300

Regarding the random effects of the models, the variance explained by the nestedness of measurements in participants for the intercept of P300 was 0.60-0.65, and regarding the slopes it was 0.04-0.72 (see Supplementary Table 4 for details on the random effects).

#### 3.3.1. Associations between P300 and mindsets

**FIGURE 3:**
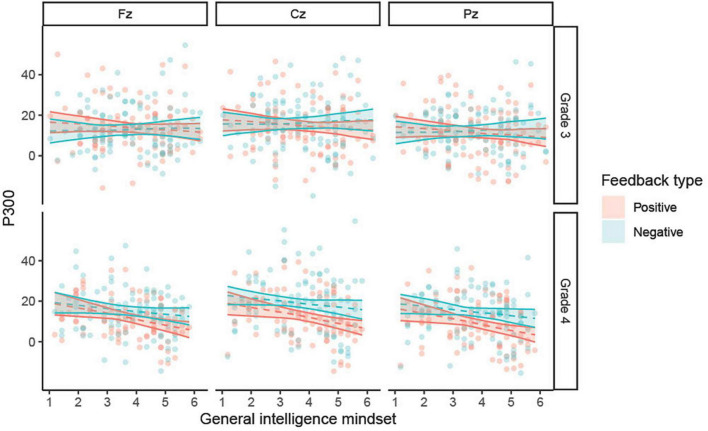
General intelligence mindset and P300 responses to positive and negative feedback in grades 3 and 4 at electrodes Fz, Cz, and Pz. Observations are shown as points, and LMM model predictions based on fixed effects are shown as dashed lines with bootstrapped 95% confidence bands.

**FIGURE 4:**
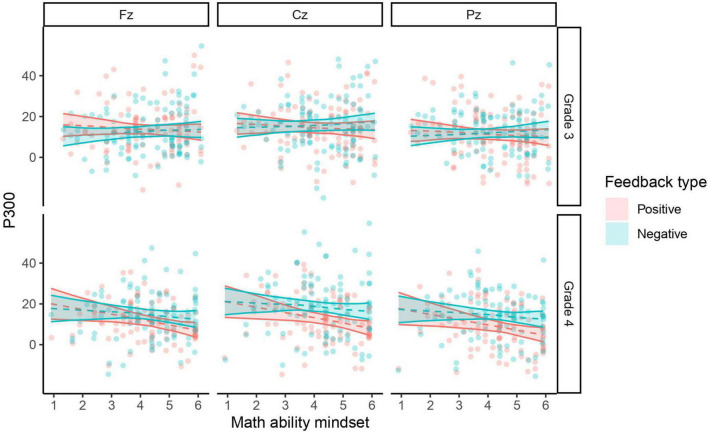
Math ability mindset and P300 responses to positive and negative feedback in grades 3 and 4 at electrodes Fz, Cz, and Pz. Observations are shown as points, and LMM model predictions based on fixed effects are shown as dashed lines with bootstrapped 95% confidence bands.

In general, the effects of GIM and MAM on P300 were both similar, although in contrast to MAM, GIM also exerted a marginal main effect on P300 (Table 3), with a higher growth mindset about general intelligence marginally associating with a smaller P300 (β = -0.10, SE = 0.08). Significant interaction effects of mindsets and feedback type as well as marginally significant interaction effects of mindsets and grade level emerged (Table 3). Simple slope comparisons revealed that the effect of mindsets (both GIM and MAM) on P300 differed between feedback types at both grade levels (*t* ≤ -2.00, *p* ≤ 0.045, for all). More specifically, a stronger growth mindset was associated with a smaller P300 in the case of positive feedback, while based on 95% confidence intervals a similar trend for the effect of GIM on negative-feedback P300 in grade 4 was observed (Table 4; see also Figures 3, 4). Additionally, the interaction effects of mindsets and grade level only approached significance, and the confidence intervals indicate that it was only in grade 4 that the effects of mindsets on P300 differed from 0 (Table 4).

**TABLE 3:** Results of linear mixed models predicting P300 amplitude.

Fixed factor	General intelligence mindset					Math ability mindset				
	***df*_*Num*_**	***df*_*Den*_**	***F***	***p***	***∈^2^_*p*_***	***df*_*Num*_**	***df*_*Den*_**	***F***	***p***	***∈^2^_*p*_***
Mindset	1	53.81	3.46	0.068	0.04	1	111.63	2.05	0.155	0.00
Feedback type	1	91.80	9.62	**0**.**003**	0.08	1	92.61	9.77	**0**.**002**	0.09
Grade level	1	92.28	0.00	0.989	0.00	1	85.89	0.01	0.926	0.00
Electrode site	2	843.55	31.86	**<0**.**001**	0.07	2	844.30	31.61	**<0**.**001**	0.07
Feedback type × electrode site	2	843.55	4.38	**0**.**013**	0.00	2	844.30	4.35	**0**.**013**	0.00
Task accuracy	1	167.20	0.04	0.833	0.00	1	166.55	0.22	0.637	0.00
Mindset × feedback type	1	715.60	10.57	**0**.**001**	0.01	1	558.80	7.79	**0**.**005**	0.01
Mindset × grade level	1	134.08	2.93	0.089	0.01	1	130.46	3.36	0.069	0.02
Feedback type × grade level	1	865.51	34.67	**<0**.**001**	0.04	1	867.19	33.71	**<0**.**001**	0.04
Feedback type × task accuracy	1	348.36	46.27	**<0**.**001**	0.11	1	339.34	42.17	**<0**.**001**	0.11
Mindset × feedback type × grade level	1	923.86	0.39	0.533	0.00	1	907.86	0.00	0.972	0.00
Task accuracy × grade level	1	118.07	2.93	0.089	0.02	1	105.84	4.72	**0**.**032**	0.03
Task accuracy × electrode site	2	843.55	11.88	**<0**.**001**	0.03	2	844.30	11.78	**<0**.**001**	0.02
Task accuracy × feedback type × grade level	1	938.98	16.35	**<0**.**001**	0.02	1	943.32	15.58	**<0**.**001**	0.02

*df_Num_* indicates the degrees of freedom numerator. *df_Den_* indicates the degrees of freedom denominator. Significant *p*-values are marked in bold.

**TABLE 4:** Effects of mindsets on positive- and negative-feedback P300 in grade 3 and 4 based on follow-up simple slope comparisons.

Predictor	Positive-feedback P300					Negative-feedback P300				
	**β**	***SE***	**95% CI**		***p***	**β**	***SE***	**95% CI**		***p***
			**LL**	**UL**				**LL**	**UL**	
GIM in grade 3	-0.10	0.08	-0.27	0.06	0.235	0.06	0.09	-0.11	0.23	0.489
GIM in grade 4	-0.26	0.08	-0.42	-0.09	**0**.**002**	-0.14	0.08	-0.30	0.02	0.086
MAM in grade 3	-0.05	0.08	-0.21	0.10	0.527	0.07	0.08	-0.08	0.23	0.376
MAM in grade 4	-0.24	0.09	-0.41	-0.07	**0**.**006**	-0.11	0.09	-0.28	0.06	0.205

Significant *p*-values are marked in bold. GIM, general intelligence mindset. MAM, math ability mindset.

#### 3.3.2. Effects of feedback type, electrode site, grade level and task accuracy on P300

Overall, both P300 models (the model including GIM as a predictor and the model with MAM as a predictor) resulted in significant main effects of feedback and electrode site and significant interaction effects between feedback type and grade level as well as between feedback type and electrode site (Table 3). *Post-hoc* tests revealed that while there was no significant difference between positive- and negative-feedback P300 in grade 3 (*t* ≥ -0.24, *p* ≥ 0.811, for both models), in grade 4, P300 was larger in case of negative feedback when compared to positive feedback (*t* ≤ -5.30, *p* < 0.001, for both models; see also Table 1). As to the effect of electrode site, negative-feedback P300 (averaged across grade levels) was larger at Cz than at Fz (*t* ≥ 5.43, *p* < 0.001, for both models) and at Pz (*t* ≥ 6.24, *p* < 0.001, for both models). Positive-feedback P300 was larger at Fz and Cz when compared to Pz (*t* ≥ 3.57, *p* ≤ 0.003, for all; see also Table 1).

Regarding the effects of participants’ accuracy on the task, both models again resulted in overall similar results. More specifically, both models revealed significant interactions between task accuracy and feedback as well as task accuracy and electrode site (Table 3). The interaction effect of task accuracy and grade level reached significance only in the model including MAM as a predictor, although it was marginal in the model including GIM as predictor (Table 3). Additionally, there was a significant three-way interaction effect of accuracy, feedback type and grade level in both models (Table 3). According to simple slope comparisons, the effect of accuracy on P300 differed between positive and negative feedback at both grade levels (*t* ≤ -3.06, *p* ≤ 0.007, for all). More specifically, higher accuracy was associated with a smaller positive-feedback P300 (see Supplementary Table 5 for the coefficients of the effects of accuracy on both feedback types at both grade levels). In the case of negative feedback, the effect of accuracy on P300 differed significantly between grade levels (*t* ≤ -2.97, *p* ≤ 0.011, for both models), with higher accuracy associated with a greater negative-feedback P300 only in grade 4 (Supplementary Table 5). As to electrode sites, the effect of accuracy on P300 (averaged across grade levels) did not differ between Fz and Cz (*t* ≥ -1.42, *p* ≥ 0.315, for all), but, in the case of positive feedback, the effect of accuracy emerged at Pz (*t* ≥ 3.32, *p* ≤ 0.006, for all; see Supplementary Table 6 for the coefficients of the effects of accuracy at each electrode site), while, in the case of negative feedback, it only emerged at Cz and Fz (*t* ≥ 3.32, *p* ≤ 0.006, for all; Supplementary Table 6).

## 4. Discussion

The main aim of the present study was to explore the development of the associations between elementary school students’ mindsets and the attentional neural processing of feedback. More specifically, we focused on the development of associations between elementary school students’ general intelligence mindset and math ability mindset and attention allocation to feedback as indicated by their P300 response elicited by both positive and negative performance-relevant feedback in an arithmetic task. We found that a more fixed mindset about both general intelligence and math ability was associated with greater attention allocation to positive feedback, with these associations being driven by the effects of mindsets on attention allocation to positive feedback in grade 4. Regarding the participants’ general intelligence mindset, a similar trend was also observed in the case of negative feedback once the children were older. Our results also indicate that the effects of both mindsets on attention allocated to feedback were marginally stronger for the participants when they were in grade 4.

### 4.1. Mindsets

At both grade levels, we found that students with a stronger growth mindset about general intelligence also displayed more of a growth mindset about math ability. This is in line with research indicating a certain intraindividual generality of mindsets regarding different domains (Lewis et al., 2021). Additionally, both mindsets showed weak to moderate stability over the two assessment points, which is in line with earlier findings on elementary school students (Pomerantz and Saxon, 2001; Kim and Park, 2021). In grade 4, a stronger growth mindset about math ability was associated with higher overall accuracy on the arithmetic task, while this association did not emerge in grade 3. This result possibly reflects the development of students’ mindsets into more coherent meaning systems during elementary school years (Kinlaw and Kurtz-Costes, 2007). The association between overall accuracy on the arithmetic task and a math ability mindset, but not a general intelligence mindset, is in line with the meta-analysis performed by Costa and Faria (2018), who concluded that students’ subject-domain-specific mindsets were more strongly associated with their achievement in the respective subject-domain than were mindsets related to another domain.

### 4.2. Associations between mindsets and P300 elicited by positive and negative feedback

Partially contradicting our expectations regarding the effect of mindsets on P300 in the case of different feedback types (H1a), we found that students with a more fixed mindset about general intelligence as well as math ability allocated greater attention to positive feedback, as indicated by a larger P300 elicited by positive feedback. These associations were driven by the effects of mindsets on positive-feedback P300 in grade 4. Regarding the participants’ general intelligence mindset, a similar trend was observed in the case of negative feedback when the children were older. The direction of these associations between mindsets and feedback-related P300 found in the present study are in line with the single earlier study on mindsets and feedback processing analyzing positive- and negative-feedback P300 separately (Mangels et al., 2006). Mangels et al. (2006) studied these associations among adults using a general knowledge task and found that a stronger fixed mindset was associated with more attention allocated to negative feedback, as indicated by a larger P300 elicited by negative feedback stimulus. Nevertheless, while the association between mindset and attention allocation to negative feedback was expected (although found to be marginal in our study), our results on the associations with attention allocated to positive feedback diverge from those of Mangels et al. (2006), as no such association emerged in their study. One possible explanation for these differences relates to the relevance of the experimental tasks to the daily lives of the participants. Mangels et al. (2006) conducted their study among undergraduates and used general knowledge questions from a variety of domains, while, in the present study on 3rd and 4th graders, we focused on the subject-domain of math. Math is a highly relevant subject-domain for elementary school students—they attend math classes regularly and consider math to be important (Tuohilampi and Hannula, 2013; McGeown and Warhurst, 2020). Therefore, in Mangels et al. (2006), it is possible that the effects of mindset on positive-feedback P300 failed to emerge because of the low relevance of the task to the daily lives of the participants. Compared to neutral stimuli, self-relevant stimuli have been shown to receive more attentional resources, as indicated by a larger P300 (Gray et al., 2004). Therefore, the greater attention allocated to feedback in the case of students holding a more fixed mindset could possibly reflect the higher self-relevance of this feedback information to them. Another possibility is that a fixed mindset may result in feedback being experienced as threatening in general. In adults, threat stimuli have been shown to elicit a larger P300 than non-threat stimuli (Wang et al., 2021), and a stronger fixed mindset among undergraduates has been shown to relate to a higher perceived threat of negative feedback (Zingoni and Byron, 2017). P300 is consistently shown to be greater for outcomes of a large magnitude than for outcomes of a small magnitude, independent of the valence of the outcome (for a review, see San Martín, 2012). Therefore, the results of the present study could be due to fixed-minded students experiencing feedback to be an outcome of greater magnitude than do growth-minded students, which is possibly a reflection of the greater self-relevance or perceived threat of the feedback stimuli for these students.

Another possible explanation concerns more general stimulus processing in stressful situations. Namely, a recent study conducted by Janssen et al. (2021) among adults found a fixed mindset to be marginally associated with a larger P300 elicited by the stop-stimulus in a go/no-go task. In an earlier study, Schroder et al. (2014) also demonstrated an association between experimentally induced mindsets and the processing of task-relevant stimuli, although they found that a fixed mindset was associated with a smaller P300 than a growth mindset. As suggested by Janssen et al. (2021), these inconsistencies regarding associations could reflect the effects of contextual factors. For example, whether individuals holding a fixed mindset learn from negative feedback seems to depend on whether the feedback they receive is contextualized as informative or evaluative (Bejjani et al., 2019). In the present study, participants’ ERPs were recorded as they completed an arithmetic task where they made errors and received performance-relevant feedback throughout the task. It is probable that the majority of students considered the feedback to be evaluative and therefore experienced the task situation as somewhat stress-inducing. In the study by Janssen et al. (2021), the participants had completed a stress-inducing arithmetic task immediately prior to data collection from the go/no-go task. Both of these studies demonstrated a similar direction for the association between mindset and attention allocation. Therefore, it is possible that these findings reflect the effect of mindsets on stimulus processing more generally when it comes to stressful situations. Nevertheless, as there was only a marginal effect on negative-feedback P300, and this only emerged in case of a general intelligence mindset, these results should be considered with caution and require replication.

The reason for our failure to find clear effects of mindsets on negative-feedback P300 is possibly due to the confounding effects of expectations regarding the negative feedback stimulus. Namely, in the case of higher accuracy in the task, the negative-feedback stimulus was presented less often and thus was probably less expected by the participant. Participants’ accuracy was associated with their P300 responses, with this association being more pronounced in grade 4 in the case of negative feedback (for a longer discussion on the effects of task accuracy, see below). It was also in grade 4 that participants’ math ability mindset was positively associated with their accuracy in the arithmetic task. Nevertheless, no stronger effect of a math ability mindset on attention allocation, when compared to a general intelligence mindset, emerged in the present study (H1b). The study by Puusepp et al. (2021) used the same experimental design and 3^rd^ grade cross-sectional data from the same sample analyzed in the present study. However, they demonstrated a marginal unique effect of a math ability mindset on the difference between P300 elicited by positive and negative feedback when controlling for the effect of general intelligence mindset. Nevertheless, the researchers focused on examining the relative effects of a general intelligence and math ability mindset and did not control for the potentially confounding effects of task accuracy. The results of the present study indicate that when it comes to attention allocated to feedback in math, it is the individual’s general growth or fixed mindset tendency, rather than the domain-specific aspects of their math ability mindset, that is associated with their brain responses (Lewis et al., 2021).

### 4.3. Development of the associations between mindsets and feedback-related P300

Regarding both general intelligence mindset and math ability mindset, we observed a marginal increase in the association between mindsets and attention allocation to feedback as the students aged (H2). Researchers have suggested that, during the first years of elementary school, students’ mindset meaning systems are still developing towards a greater coherence (Kinlaw and Kurtz-Costes, 2007). More specifically, with their cross-sectional data, Kinlaw and Kurtz-Costes (2007) demonstrated that 2nd graders’ goal-orientation is more in line with their mindsets when compared to preschoolers. Our longitudinal results demonstrating a marginal increase in the association between students’ mindsets and their attention allocation to feedback could reflect a similar developmental trend. It is possible that the one year between measurement points used in the present study was too short a period to observe a significant developmental effect. Nevertheless, as the effect demonstrated in our study was only marginal, our results should be considered with caution and require replication.

### 4.4. P300 responses: Effects of grade level, feedback, electrode site and task accuracy

When analyzing the development of the amplitude of P300 responses, we found no overall differences between 3rd and 4th grade students. Nonetheless, a large-scale study by Dinteren et al. (2014) indicates that the amplitudes of P300 responses (elicited by auditory stimulus) continue to increase until the age of 21. It is possible that one year is a too short period to catch such a developmental trend. Additionally, Dinteren et al. (2014) used a different paradigm than that employed by the present study.

When investigating the neural processing of positive and negative feedback, we found that the P300 responses elicited by such feedback did not differ among students in grade 3, but, in grade 4, greater P300 amplitudes were elicited by negative feedback than by positive feedback. Earlier findings on the effects of outcome valence on P300 have been mixed (children: e.g., Buritica et al., 2018; Arbel, 2020; adults: e.g., Butterfield and Mangels, 2003; Hajcak et al., 2005; Ernst and Steinhauser, 2012; for a review, see San Martín, 2012). At least among adults, a greater P300 amplitude has been shown to associate with the heightened psychological significance of the stimulus (Gray et al., 2004). Therefore, it is possible that, as students age, the difference between the subjective significance of positive and negative feedback in math becomes greater for them. Moreover, the greater P300 elicited by negative rather than positive feedback in grade 4 possibly reflects the increased salience of negative performance feedback in math compared to positive feedback.

Regarding electrode sites, we found negative-feedback P300 to be greatest at the central site and positive-feedback P300 to be larger at the frontal and central sites than at the parietal site. In line with this result, Wu and Zhou (2009) and Mangels et al. (2006) found P300 to be maximal at the central site, although Ferdinand et al. (2016) produced conflicting results. As to participants’ overall accuracy in the task, we found higher accuracy to be associated with a smaller positive-feedback P300 at the parietal site. As to negative feedback, it was only in grade 4 that higher accuracy was associated with a larger negative-feedback P300, with this effect emerging only at the frontal and central site. Earlier studies among adults (Hajcak et al., 2005; Wu and Zhou, 2009; for a review, see San Martín, 2012) as well as children (Ladish and Polich, 1989; but see also Ferdinand et al., 2016) have found P300 amplitude to be sensitive to the probability of the stimulus, with expected stimuli eliciting smaller P300 responses compared to unexpected stimuli. In the case of higher accuracy in the task, the negative-feedback stimulus is presented less frequently and thus is possibly less expected by the participant. Therefore, the positive association between task accuracy and negative-feedback P300 as well as the negative association between task accuracy and positive-feedback P300 probably reflect the effect of expectations about the negative and positive feedback stimulus on P300.

### 4.5. Limitations and practical implications

There are several limitations that should be borne in mind when interpreting the results of the present study. First, in contrast to positive feedback, negative feedback included a feedback sound. Although this difference between the two feedback types somewhat limits the comparison of attention allocation to positive and negative feedback, we prioritized the clarity of the feedback type for the participants. Therefore, we used the feedback sound in the case of negative feedback to ensure that the participants were clearly aware that they had made an error. Therefore, it is possible that the confounding effect of the negative feedback sound explains why we only observed a marginal effect of mindset on negative-feedback P300, while the effects on positive-feedback P300 were more pronounced.

Second, just as overall task accuracy varied between participants, so did the frequency of the receipt of negative and positive feedback. As stated earlier, the probability of the stimulus affects the elicited P300 response (Hajcak et al., 2005; Wu and Zhou, 2009), and therefore the findings of the present study might be confounded by the effect of stimulus probability on attention allocation, although we did account for task accuracy in our models. While predetermined equal frequencies for negative and positive feedback could have been used, we prioritized participants’ experience of the truthfulness of the feedback and therefore presented them with the actual feedback regarding their responses. In future, algorithms could be used to continuously adjust the difficulty of the arithmetic task to the participant’s performance, ensuring relatively equal levels of task accuracy among the participants. This would enable the avoidance of the potentially confounding effects of the frequency of both feedback stimuli and therefore allow the effects of mindsets to be assessed more accurately.

Third, in the case of negative feedback in the present study, performance-relevant (information on the accuracy of the response) and corrective feedback (information on the correct response) were presented simultaneously. In future, these two feedback types should be presented sequentially, enabling the inspection of the effects of mindsets on attention allocation to both performance-relevant as well as corrective feedback.

Fourth, we did not assess participants’ subjective experiences during the experiment. Future studies could ask participants to report, for example, their level of enjoyment or anxiety during the task, as inspecting associations between these self-reports, mindsets, and P300 would enable more informative inferences to be made regarding the associations between mindsets and attention allocation.

Finally, students’ mindsets were assessed using self-report measures. However, research has shown that the use of self-report questionnaires among young children might be problematic, as such children may experience difficulties understanding the questions and engaging in the self-reflection necessary to answer the questions (Borgers et al., 2000). Nevertheless, in the present study, we used different sized circles that mapped to a 6-point scale to help the participants more easily report their agreement with the statements. We also used example statements to ensure the participants understood how to indicate their agreement with the questionnaire items. Additionally, in the 3rd grade, the questions were read out by the researcher one by one. Moreover, at both grade levels, the two-factor models of correlated factors fit the mindset data well (see Supplementary Table 1), and the internal consistencies of the scales ranged from acceptable to very good. In addition, we demonstrated that both mindsets were correlated with multiple theoretically relevant measures at both grade levels (see Supplementary Table 2).

As to the practical implications of our results, the present study is rather limited, as it only explored the associations between mindsets and performance-relevant feedback. The present findings and earlier research demonstrating the role of mindsets regarding perceptions of feedback (Zingoni and Byron, 2017) suggest that similar feedback can be perceived differently by individual learners, possibly leading to more adaptive behaviors among some students and less adaptive reactions among others. In addition, earlier research has demonstrated the importance of the type of feedback children receive regarding their learning behavior and the development of their mindsets. More specifically, receiving feedback focused on one’s fixed qualities (person-oriented feedback) seems to lead to lower task persistence, endorsement of performance goals, and a fixed mindset (e.g., Mueller and Dweck, 1998; for a review, see Haimovitz and Dweck, 2017). Feedback regarding one’s effort and strategies (process-oriented feedback), on the other hand, is suggested to lead to higher task persistence and endorsement of learning goals and growth mindset (e.g., Mueller and Dweck, 1998; Gunderson et al., 2013; for a review, see Haimovitz and Dweck, 2017). In the current study, the feedback was neither person- nor process-oriented; rather, it provided information solely about the participants’ performance on each trial. It is possible that students holding a more fixed mindset tend to experience such neutral—neither process- nor person-oriented—feedback as more self-relevant than do growth-minded students. Hence, in contrast to growth-minded students, fixed-minded students may experience exclusively performance-relevant feedback as rather similar to person-oriented feedback—as indicative of their fixed ability. This highlights the need for awareness of the potential impacts of feedback on students, as well as the context of feedback, which might be perceived as more threatening by students holding a fixed mindset. It is plausible that providing process-oriented feedback, instead of feedback containing information solely on the current performance, would guide fixed-minded students to view their current performance as part of a long-term learning process and thus support these students in developing a more growth-oriented mindset (Haimovitz and Dweck, 2017; see Wisniewski et al., 2020 for a meta-analysis on the effects of feedback type on student outcomes). Nevertheless, as the current study is limited to performance-relevant feedback, it remains for future research to explore whether the associations between mindsets and attention allocation to process-oriented feedback differ from the associations found in the present study. Additionally, our results emphasize the need to examine the associations between mindsets and the attentional processing of stimuli more generally; moreover, they highlight the importance of exploring the potential effects of contextual factors.

In sum, the findings of the present study demonstrated the association between elementary school students’ fixed mindsets about general intelligence and math ability and greater attention allocated to positive feedback in an arithmetic task, with a similar trend identified for the effect of a general intelligence mindset in the case of negative feedback among older children. These results, although tentative and with small effect sizes, suggest that the perspective should be widened to explore the effects of students’ mindsets on their experiences more generally rather than focusing primarily on situations involving setbacks.

## Data availability statement

The raw data supporting the conclusions of this article will be made available by the authors, without undue reservation.

## Ethics statement

The studies involving human participants were reviewed and approved by the University of Helsinki Ethical Review Board. Written informed consent to participate in this study was provided by the participants’ legal guardian/next of kin.

## Author contributions

IP, MH, TK, EK, SL, and KT planned the research design. IP collected the data and wrote the manuscript. IP, TL, and TT pre-processed the data and conducted the analyses. All authors contributed to the article and approved the submitted version.

## Footnotes

1 As the Implicit Theories of Intelligence Scale was originally recommended for children of 10 years or older (Dweck, 2000, p. 177), we used confirmatory factor analysis to confirm that these two mindsets (GIM and MAM) formed two distinct but correlated factors at both grade levels (see Supplementary Table 1). Additionally, we examined whether the associations between mindsets and theoretically related constructs emerged in the present sample (see Supplementary Table 2).
