# Supplementary material for: Development of associations between elementary school students’ mindsets and attentional neural processing of feedback in an arithmetic task
Source: Front Psychol. 2023 Mar 14;14:1155264. doi: 10.3389/fpsyg.2023.1155264 (PMC10043393; doi:10.3389/fpsyg.2023.1155264)
Supplement: Supplementary file 1 [file Table_1.docx]

Supplementary Material

**Table 1**

*Model Fit Indices of Confirmatory Factor Analyses with Two Correlated Factors (General Intelligence Mindset and Math Ability Mindset)*

|  | χ2 | df | CFI | TLI | RMSEA | SRMR |
| --- | --- | --- | --- | --- | --- | --- |
| Two-factor model in grade 3 | 28.91 | 19 | .960 | .941 | .073 | .052 |
| Two-factor model in grade 4 | 21.87 | 19 | .992 | .988 | .039 | .045 |

*Note.* All standardized factor loadings ranged between .53 and .93 and were significant at *p <* .001.
In grade 3, the correlation between factors was .54, *p* < .001, and in grade 4 it was .79, *p* < .001.

**Table 2**

*Correlations Between Mindsets and Theoretically Relevant Constructs from Grades 3 and 4*

| Variable | *n* | 1 | 2 | 3 | 4 | 5 | 6 | 7 | 8 |
| --- | --- | --- | --- | --- | --- | --- | --- | --- | --- |
| 1. Teacher-reported student reactions to challenges in grade 3 | 92 | − |  |  |  |  |  |  |  |
| 2. Challenge preference in math in grade 3 | 98 | .13 | − |  |  |  |  |  |  |
| 3. Effort beliefs in grade 3 | 99 | .22^*^ | .23^*^ | − |  |  |  |  |  |
| 4. General intelligence mindset in grade 3 | 99 | .26^*^ | .07 | .20^*^ | − |  |  |  |  |
| 5. Math ability mindset in grade 3 | 99 | .25^*^ | .32^**^ | .29^**^ | .43^***^ | − |  |  |  |
| 6. Challenge preference in math in grade 4 | 94 | .23^*^ | .34^***^ | .20^†^ | .18^†^ | .27^**^ | − |  |  |
| 7. Effort beliefs in grade 4 | 96 | .16 | .19^†^ | .35^***^ | .14 | .50^***^ | .39^***^ | − |  |
| 8. General intelligence mindset in grade 4 | 96 | .18^†^ | .23^*^ | .33^**^ | .22^*^ | .26^**^ | .18^†^ | .41^***^ | − |
| 9. Math ability mindset in grade 4 | 96 | .18^†^ | .22^*^ | .37^***^ | .23^*^ | .35^***^ | .28^**^ | .60^***^ | .67^***^ |

*Note.* ^†^ *p* < .10; * *p* < .05; ** *p* < .01; *** *p* < .001. Higher effort belief values indicate stronger beliefs about the utility of effort in enhancing one’s ability; teacher-reported student reactions to challenges (1 – very negative … 4 – very positive); behavioral assessment of challenge preference in math (dichotomous: 0 – easy task in order to succeed, 1 – challenging task in order to learn).

**Table 3**
*Results of Linear Mixed Models on P300, Including Background Variables and Task Accuracy*

| Fixed factor | Model 1 | | | | | Model 2 | | | | |
| --- | --- | --- | --- | --- | --- | --- | --- | --- | --- | --- |
|  | *df_Num_* | *df_Den_* | *F* | *p* | *df_Num_* | | *df_Den_* | *F* | *p* |  |
| Feedback type | 1 | 94.99 | 8.58 | **.004** | 1 | | 95.51 | 11.18 | **.001** |  |
| Grade level | 1 | 100.30 | 3.84 | .053 | 1 | | 98.39 | 0.00 | .987 |  |
| Electrode site | 2 | 874.79 | 29.97 | **< .001** | 2 | | 870.50 | 33.50 | **< .001** |  |
| Feedback type × grade level | 1 | 889.23 | 20.65 | **< .001** | 1 | | 886.17 | 26.95 | **< .001** |  |
| Feedback type × electrode site | 2 | 874.79 | 4.57 | **.011** | 2 | | 870.50 | 4.67 | **.010** |  |
| Electrode site × grade level | 2 | 874.79 | 0.40 | .673 | 2 | | 870.50 | 1.49 | .226 |  |
| Task accuracy |  |  |  |  | 1 | | 178.68 | 0.11 | .740 |  |
| Task accuracy × feedback type |  |  |  |  | 1 | | 384.45 | 52.54 | **< .001** |  |
| Task accuracy × electrode site |  |  |  |  | 2 | | 870.50 | 9.86 | **< .001** |  |
| Task accuracy × grade level |  |  |  |  | 1 | | 124.66 | 2.19 | .142 |  |
| Task accuracy × feedback type × electrode site |  |  |  |  | 2 | | 870.50 | 0.38 | .685 |  |
| Task accuracy × feedback type × grade level |  |  |  |  | 1 | | 963.22 | 16.58 | **< .001** |  |

*Note*. *df_Num_* indicates the degrees of freedom numerator. *df_Den_* indicates the degrees of freedom denominator.
Significant p-values are marked in bold.

**Table 4**
*Random Effects of Linear Mixed Models on P300*

| Random effect | General intelligence mindset model | Math ability mindset model |
| --- | --- | --- |
|  | *SD* | *SD* |
| Intercept | .80 | .77 |
| Slope of mindset | .19 | .21 |
| Slope of grade level | .82 | .85 |
| Slope of feedback type | .57 | .56 |
| Residual | .53 | .53 |

**Table 5**
*Effects of Task Accuracy (Averaged Across Electrode Sites) on Positive- and Negative-Feedback P300 at Both Grade Levels Based on Follow-up Simple Slope Comparisons*

|  | Positive-feedback P300 | | | | | | Negative-feedback P300 | | | | |  |
| --- | --- | --- | --- | --- | --- | --- | --- | --- | --- | --- | --- | --- |
| Grade level | *β* | *SE* | 95% CI | | *p* | *β* | | *SE* | 95% CI | | *p* | |
|  |  |  | LL | UL |  |  | |  | LL | UL |  | |
| Grade 3 | −.17 / −.17 | .08 | −.34 / −.33 | −.01 / −.01 | **.043** / **.037** | .03 / .02 | | .09 / .08 | −.14 / −.15 | .20 / .18 | .729 / .812 | |
| Grade 4 | −.15 / −.10 | .08 | −.30 / −.26 | .01 / .06 | .058 / .221 | .34 / .36 | | .08 | .18 / .20 | .50 / .53 | < **.001** / **<** **.001** | |

*Note.* Standardized coefficients, standard errors and confidence intervals from simple slope comparisons based on the LMM including GIM as predictor are presented before the forward slash, while the ones based on the LMM including MAM as predictor are shown after the forward slash. In case the standard errors did not differ between the models, only one value is reported. Significant p-values are marked in bold.

**Table 6**
*Effects of Task Accuracy (Averaged Across Grade Levels) on Positive- and Negative-Feedback P300 at Fz, Cz and Pz Based on Follow-up Simple Slopes Comparisons*

|  | Positive-feedback P300 | | | | | Negative-feedback P300 | | | | |
| --- | --- | --- | --- | --- | --- | --- | --- | --- | --- | --- |
| Electrode site | *β* | *SE* | 95% CI | | *p* | *β* | *SE* | 95% CI | | *p* |
|  |  |  | LL | UL |  |  |  | LL | UL |  |
| Fz | −.08 / −.06 | .07 | −.21 / −.18 | .05 / .07 | .228 / .347 | .26 / .27 | .07 | .13 / .13 | .40 / .40 | < **.001** / **<** **.001** |
| Cz | −.13 / −.11 | .07 | −.27 / −.24 | .00 / .02 | .059 / .097 | .21 / .21 | .07 | .07 / .08 | .34 / .35 | **.002** / **.002** |
| Pz | −.26 / −.24 | .07 | −.39 / −.37 | −.13 / −.11 | < **.001** / **<** **.001** | .08 / .09 | .07 | −.05 / −.05 | .22 / .22 | .245 / .191 |

*Note.* Standardized coefficients and confidence intervals from simple slope comparisons based on the LMM including GIM as predictor are presented before the forward slash, while the ones based on the LMM including MAM as predictor are shown after the forward slash. As the standard errors did not differ between the models, only one value is reported. Significant p-values are marked in bold.
